# Role of Ginkgolides in the Inflammatory Immune Response of Neurological Diseases: A Review of Current Literatures

**DOI:** 10.3389/fnsys.2020.00045

**Published:** 2020-07-31

**Authors:** Chunrong Li, Kangding Liu, Shan Liu, Qiaolifan Aerqin, Xiujuan Wu

**Affiliations:** Department of Neurology, Neuroscience Center, The First Hospital of Jilin University, Jilin University, Changchun, China

**Keywords:** ginkgolides, inflammatory immune response, neurological diseases, multiple sclerosis, Guillain–Barré syndrome

## Abstract

The inflammatory immune response (IIR) is a physiological or excessive systemic response, induced by inflammatory immune cells according to changes in the internal and external environments. An excessive IIR is the pathological basis for the generation and development of neurological diseases. Ginkgolides are one of the important medicinal ingredients in *Ginkgo biloba*. Many studies have verified that ginkgolides have anti-platelet-activating, anti-apoptotic, anti-oxidative, neurotrophic, and neuroimmunomodulatory effects. Inflammatory immunomodulation is mediated by inhibition of the mitogen-activated protein kinase (MAPK) and nuclear factor-kappa B (NF-κB) signaling pathways. They also inhibit the platelet-activating factor (PAF)-mediated signal transduction to attenuate the inflammatory response. Herein, we reviewed the studies on the roles of ginkgolides in inflammatory immunomodulation and suggested its potential role in novel treatments for neurological diseases.

## KEY POINTS

1. Ginkgolides have inflammatory immunomodulation effects, which are mediated by inhibition of the MAPK and TLR/MyD88/NF-κB signaling pathways.
2. TLR/MyD88/NF-κB signaling pathways are involved in the pathogenesis of some neurological diseases.
3. However, there are currently no comprehensive reviews about the regulatory effects of ginkgolides on the IIR.
4. Ginkgolides may represent a potential therapeutic target for neurological disorders in the future.

## Introduction

The inflammatory immune response (IIR) is a physiological or excessive systemic response, induced by inflammatory immune cells based on the changes in the internal and external environments (Han et al., 2018). Inflammatory immune cells such as macrophages (MΦ), T lymphocytes, dendritic cells (DCs), some nonimmune cells, inflammatory immune cytokines, and related receptor signal transduction pathways are involved in the mechanisms underlying excessive IIR (Han et al., 2018). An excessive IIR is the pathological basis for the generation and development of neurological diseases, especially neurodegenerative and/or neuroimmune diseases, and ischemic cerebrovascular diseases (Ritzel et al., 2018; Voet et al., 2019). Regulating an excessive IIR has become a novel therapeutic target for neurological diseases. Ginkgolides are isolated and purified from the leaves of *Ginkgo biloba*. The ginkgo leaf extracts commonly contain flavonoids such as quercetin, kaempferol, myricetin, and terpene trilactone (Al-Adwani et al., 2019). The extracted terpene trilactone includes ginkgolide A (GA), B, C, M, J, and K and bilobalide (BB; Huang et al., 2014). As early as 1985, Braquet et al. discovered that ginkgolides, particularly GB, are platelet-activating factor (PAF) receptor (PAFR) antagonists, which contribute to the prevention of platelet aggregation and thrombosis (Braquent, 1985). The neuromodulatory effects of ginkgolides include promoting secretion of neurotrophic factors, anti-oxidant effects, increasing cerebral blood flow and circulation, modifying neurotransmission, and providing protection against apoptosis (Bastianetto et al., 2000; Zheng et al., 2000; Wang and Chen, 2005; Tchantchou et al., 2009; Ribonnet et al., 2011; Wei et al., 2017; Table 1). The regulatory effects of ginkgolides on IIR have recently been revealed. Ginkgolides can regulate IIR *via* PAF-mediated signal transduction, mitogen-activated protein kinase (MAPK), and toll-like receptor/myeloid differentiation primary response 88/nuclear factor-kappa B (TLR/MyD88/NF-κB) signaling pathways. Mediating an excessive IIR is a novel therapeutic target for neurological diseases, especially neurodegenerative diseases, ischemic cerebrovascular diseases, and/or neuroimmune diseases, but there are currently no comprehensive reviews on this topic. We herein summarize the articles about the effects of ginkgolides on the IIR, and we suggest a potential role for ginkgolides as a novel treatment for neurological disorders.

**Table 1:** Pharmacological characteristics of ginkgolides.

Pharmacological characteristics	Possible mechanisms	References
Anti-PAF	Competitively inhibits the binding of PAFR to ligands.	Gui et al. (2007) and Maerz et al. (2011)
Anti-apoptotic	Regulates anti-apoptotic protein Suppresses p-SAPK/JNK activation, reactive oxygen species, mitochondrial pro-apoptotic factors, PARP, and cytochrome *c* release.	Ahlemeyer et al. (1999) and Gu et al. (2012)
Anti-oxidative	Interferes with production of free oxygen radicals Protects the decrease in hippocampal Ca^2+^/calmodulin-dependent protein kinase II activity.	Zalewska et al. (1996) and Pietri et al. (1997)
Neurotrophic effect	Up-regulates the expression of BDNF.	Wei et al. (2017)
Neuroimmunomodulatory effect	Inhibits TNF-α, IL-6, IL-1β and suppresses TLR4, NF-κB gene expressions.	Hu et al. (2011)

*Note. PAF, platelet-activating factor; PAFR, platelet-activating factor receptor; PARP, poly (ADP-ribose) polymerase; p-SAPK, p-stress-activated protein kinase; JNK, c-Jun N-terminal kinase; BDNF, brain-derived neurotrophic factor; TNF-α, tumor necrosis factor α; IL, interleukin-6; TLR, toll-like receptor; NF-κB, nuclear factor-kappa B*.

## Chemical and Pharmacological Characteristics of Ginkgolides

### Chemical Characteristics of Ginkgolides

Ginkgolides consist mainly of diterpenes and sesquiterpenes, which are the only natural substances with tertiary butyl functional groups [–C17 (CH3)_3_]. As early as the 1930s, scholars had extracted and separated active components from *Ginkgo biloba* leaves (Strømgaard and Nakanishi, 2004). GA, GB, and BB in *G. biloba* were separately measured in the root, stem, and leaf by high-performance liquid chromatography in 1997 (Lu et al., 2017). The results demonstrated that quantities of GA, GB, and BB are high in the roots and leaves. Recently, GA, GB, GC, and BB have been further measured in the cortex and xylem of roots and branches (Lu et al., 2017). The diterpenoid lactones of ginkgolides have a unique 20-carbon skeleton structure, embedded with a tertiary butyl rarely found in natural compounds, and have a rigid skeleton formed by six five-membered rings, A–F. The diterpenoid lactones of *G. biloba* differ only in the number and position of hydroxyl groups, which can be converted into each other under certain conditions.

### Platelet-Activating Factor-Mediated Signal Transduction in the Regulation of Inflammatory Immune Response of Ginkgolides

**Figure 1:**
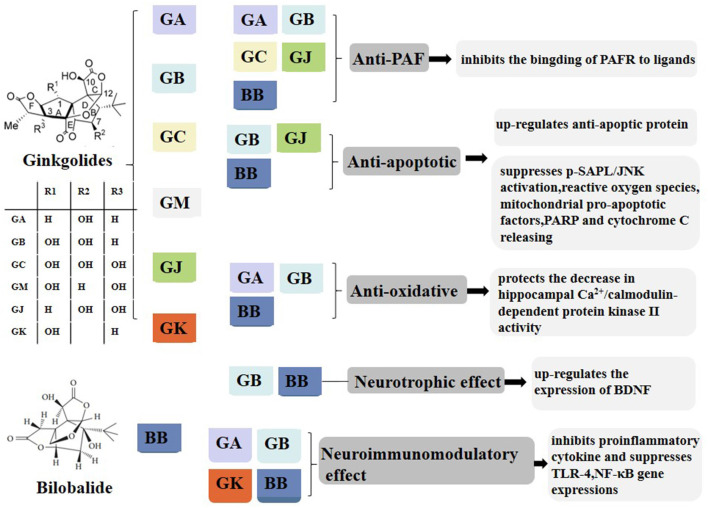
Composition and pharmacological properties of ginkgolides. Ginkgolides are isolated and purified from the leaves of *Ginkgo biloba*. The ginkgo leaf extracts commonly contain flavonoids such as quercetin, kaempferol, myricetin, and terpene trilactone. The extracted terpene trilactone include ginkgolide A (GA), B, C, M, J, and K and bilobalide (BB). Ginkgolides have anti-PAF, anti-apoptotic, anti-oxidative, neurotrophic, and neuroimmunomodulatory effects. GA, ginkgolides A; BB, bilobalide; PAF, platelet-activating factor; PAFR, platelet-activating factor receptor; p-SAPK/JNK, phospho-stress-activated protein kinase/c-Jun N-terminal kinase; PARP, poly (ADP-ribose) polymerase; BDNF, brain-derived neurotrophic factor; TLR4, toll-like receptor-4; NF-κB, nuclear factor-kappa B.

**Figure 2:**
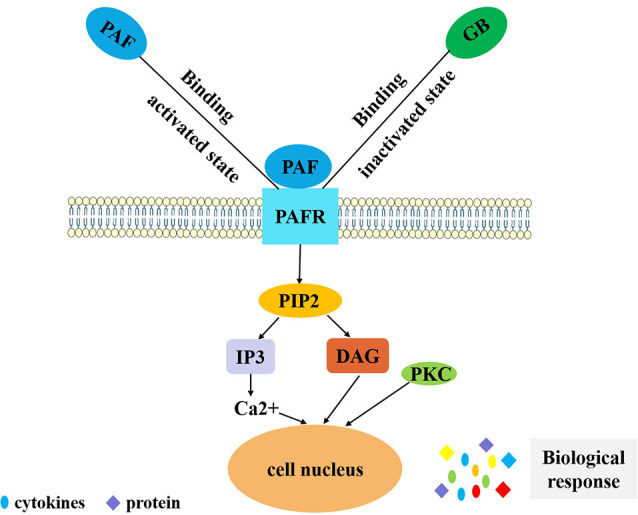
PAF-mediated signal transduction of ginkgolides in the regulation of IIR. PAF binds to PAF receptor *in vivo*, and coupled with G protein, phospholipase C is activated and has an effect on phosphatidylinositol 4,5-bisphosphate. Phosphatidylinositol bisphosphate breakdown produces inositol triphosphate and diglyceride. Inositol triphosphate can induce intracellular calcium concentration increases and diglyceride can activate PKC. Finally, it exerts a biological effect by secreting cytokines and proteins. GB competitively inhibits PAF binding to PAFR in order to reduce the above series of reactions and play an anti-inflammatory role. PAF, platelet-activating factor; IIR, inflammatory immune response; PKC, protein kinase C; GB, ginkgolide B.PAF, platelet-activating factor; PAFR, platelet-activating factor receptor; GB, ginkgolide B; PIP2, phosphatidylinositol 4,5-bisphosphate; IP3, inositol 1,4,5-triphosphate; DAG, diacylglycerol; PKC, protein kinase C; IIR, inflammatory immune response.

Pharmacological studies of ginkgolides are extensive (Table 1, Figure 1). Ginkgolides are natural PAFR antagonists that selectively and competitively antagonize PAF-induced platelet aggregation (Gui et al., 2007). It is well established that PAF signaling plays a pivotal role in the initiation and progression of inflammatory and thrombotic reactions, as well as in the cross talk between them (Stafforini et al., 2003). PAF is a lipid mediator of inflammation and has important functions in acute and chronic inflammation, emerging as an important factor in neural injury, such as ischemia/reperfusion (I/R) injury, stroke, inflammation, and multiple sclerosis (MS; Bellizzi et al., 2016; Wang et al., 2018). PAF works by binding to a unique G-protein-coupled, seven-transmembrane receptor, which contains an intronless protein coding region and activates multiple intracellular signaling pathways (Deng et al., 2019). The PAFR is considered to regulate all PAF actions through humoral, autocrine, and/or paracrine mechanisms. Kinases and phospholipases whose activation is induced by PAF include MAPK, protein kinase C (PKC), phosphatidylinositol 3-kinase (PI3K), protein tyrosine kinases, G-protein-coupled receptor kinase, and multiple intracellular signal transducers (Ishii and Shimizu, 2000). In addition, PAF regulates the expressions of interleukin (IL)-1, IL-6, IL-8, and pleiotropic cytokines (Hamel-Côté et al., 2019a,b). As an inflammatory factor, PAF plays an important role in many pathological conditions. It is remarkable that PAF is synthesized and released in both acute and chronic inflammatory animal models. PAF and PAF-like lipids bind to PAFR, which triggers a variety of intracellular signaling cascades and induces functional responses by PAFR-bearing cells, further initiating or amplifying inflammatory, thrombotic, or apoptotic events (Maerz et al., 2011). Thus, blocking PAFR signaling could possibly inhibit inflammation or ischemic injury. There is increasing evidence that GB protects against neural damage in a variety of circumstances and has beneficial effects on circulatory and inflammatory conditions due to pathophysiological effects of PAF (Golino et al., 1993). As an antagonist of the G-protein-coupled PAFR, GB is widely present on pivotal target cells of the inflammatory, immune, and hemostatic systems, and it competitively inhibits PAFR ligand binding (Gui et al., 2007; Maerz et al., 2011; Figure 2). Tran and colleagues investigated the roles of PAFR in the abnormal behaviors induced by phencyclidine (PCP) in mice, and they found that treatment with PCP resulted in a virtual increase in nuclear translocation of NF-κB p65 and deoxyribonucleic acid (DNA) binding activity. These findings indicate that levels of the pro-inflammatory molecule NF-κB are increased through up-regulation of PAFR. They also found that GB significantly attenuates abnormal behaviors such as depression, sociability and cognitive impairment, and behavioral sensitization induced by PCP, in PAFR knockout mice. Moreover, GB attenuates PCP-induced increases in NF-κB p65 nuclear translocation and DNA binding activity (Tran et al., 2018). It was proposed for the first time that PAF/PAFR mediates dopaminergic degeneration *via* an NF-κB-dependent signaling process (Kim et al., 2013). Depletion of the PAFR gene, or GB, which itself is a PAFR antagonist, significantly attenuates the increase in NF-κB DNA binding activity (Kim et al., 2013). GB has also been shown to ameliorate colonic inflammation and decrease tumor number and load in mice, through the assessment of disease activity indexes, histological injury scores, leukocyte infiltration, and expression of pro-inflammatory cytokines such as tumor necrosis factor-α (TNF-α), IL-1β, and IL-6 (Sun et al., 2015). PAF regulates cytokines, which stimulates leukotriene synthesis and is associated with the pathogenesis of inflammatory processes (Maclennan et al., 2002). The PAFR is also involved in the microglial polarization modulatory effects of GB on increasing M2 signature gene expression, reducing M1 gene expression, increasing transforming growth factor-β (TGF-β) and IL-10 secretion, and decreasing IL-6 and TNF-α (Shu et al., 2016). Both GA and GB dose dependently inhibit the production of pro-inflammatory cytokines, such as TNF-α and IL-1, in lipopolysaccharide (LPS)-stimulated rat microglial cultures (Li et al., 2017).

### Ginkgolides Regulate Mitogen-Activated Protein Kinase Signaling Pathways in the Inflammatory Immune Response

The effects of ginkgolides in inflammation and immunomodulation are gradually recognized. Administration of GB inhibits TNF-α, IL-6, and IL-1β production and suppresses TLR4 and NF-κB gene expression in an intracerebral hemorrhagic rat model (Hu et al., 2011). MAPK signaling is important for adjusting and controlling the structure and function of eukaryotic cells by transmitting signals from the cell membrane to the nucleus in response to a variety of extracellular stimuli, including neurotransmitters, hormones, inflammatory factors, viruses, growth factors, and inducer of oxidative stress (Elbirt et al., 1998; Sun and Nan, 2016). In MΦ and DC, p38 MAPK is activated by TLR and promotes the secretion of various pro-inflammatory and T cell polarization factors, such as TNF-α, interferon-γ (IFN-γ), IL-1β, IL-12, IL-6, and IL-23 (Aicher et al., 1999; Kikuchi et al., 2003). TLRs activate innate immunity through the early identification of pathogenic-associated molecular patterns in pathogens (Paul et al., 2018). These receptors also regulate adaptive immunity through up-regulating the expression of co-stimulatory molecules on the antigen presenting cell surface and the secretion of inflammatory cytokines, providing the second signal for T lymphocyte activation and inducing T lymphocyte differentiation. In T cells, p38 MAPK is activated by T cell receptor signaling, cytokines, and histamine (Berenson et al., 2006; Paul et al., 2018). c-Jun N-terminal kinase (JNK) and p38 are activated by a large number of immune receptors such as TLRs, TNFR, and IL-1R (Huang et al., 2009). JNK-mediated integration of T cell receptor and costimulation signals play a role in the stress-activated MAPK pathways in immune responses (Su et al., 2001). Coordinate immune response is one of the primary functions of stress-activated MAPK. It has been shown that pharmacological inhibition of p38 and JNK pathways is effective in treating or alleviating various inflammatory conditions (Kumar et al., 2003; Manning and Davis, 2003; Jeffrey et al., 2007).

**Figure 3:**
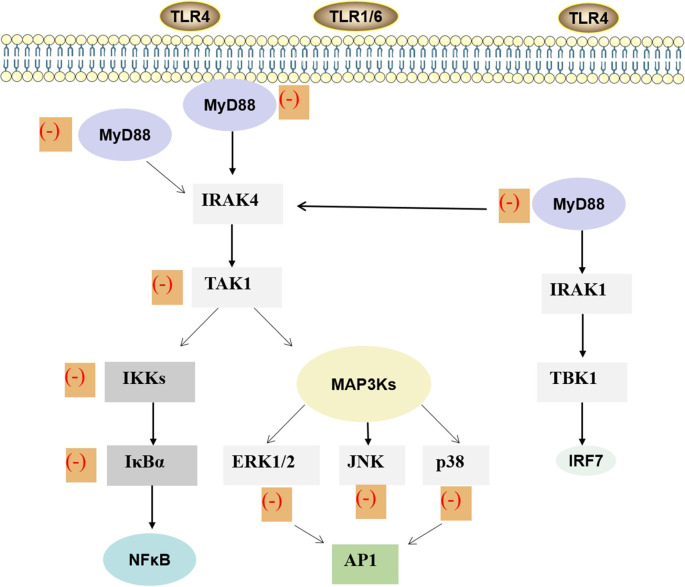
MAPK and NF-κB signaling pathway of ginkgolides in the regulation of IIR. Lipopolysaccharide stimulates the dimerization of TLR4 and activates the conserved MAPK tertiary kinase cascade through MyD88, interleukin-1 receptor-associated kinase, which leads to the activation of transcription factors. Finally, it promotes the expression of inflammatory factors in target cells, thus participating in the inflammatory reaction process induced by lipopolysaccharide. As shown in the figure, ginkgolides have effects on MAPK and TLRs/MyD88/NF-κB signaling transduction pathway. It can down-regulate MyD88, transforming growth factor-β-activated kinase-1, IκB kinases, IκBα, extracellular signal-regulated protein kinase 1/2, extracellular signal-regulated JNK, and p38, ultimately reducing inflammation. MAPK, mitogen-activated protein kinase; NF-κB, nuclear factor-kappa B; IIR, inflammatory immune response; MyD88, myeloid differentiation primary response 88; TLR, toll-like receptor.TLR, toll-like receptor; MyD88, myeloid differentiation primary response 88; IRAK, interleukin-1 receptor-associated kinase; TAK1, transforming growth factor-β-activated kinase-1; IKK, IκB kinases; MAP3K, mitogen-activated protein kinase kinase kinase; ERK, extracellular signal-regulated protein-1; JNK, c-Jun amino-terminal kinase; NF-κB, nuclear factor-κB; AP1, activator protein-1; TBK1, TANK-binding kinase 1; IRF, interferon regulatory factor; IIR, inflammatory immune response.

In order to investigate whether *G. biloba* extract EGb761, which mainly contains flavonoids and terpene lactones, could reduce cerebral p-Tau levels and prevent Alzheimer’s disease pathogenesis, human P301S tau mutant transgenic mice were fed with this compound for 5 months (Qin et al., 2018). It was found that the mouse cognitive function was improved, synaptophysin loss was attenuated, the cAMP response element binding protein phosphorylation in the mouse brain was recovered, and the p-Tau protein was decreased after treatment with EGb761 (Qin et al., 2018). Moreover, long-treatment with EGb761 also inhibited the activation of p38-MAPK and glycogen synthase kinase 3 in tau-transgenic mouse brains, the two key enzymes generating p-Tau. These all suggested that EGb761, especially the components of GA, BB, and flavonoids, enhanced autophagy, increased the degradation of phosphorylated tau in neurons, and reduced the generation of phosphorylated tau by inhibiting the activity of p38 MAPK and glycogen synthase kinase 3 (Qin et al., 2018). In addition, the neuroprotective effects of BB on cerebral ischemia and reperfusion injury are also associated with inhibition of pro-inflammatory mediator production and down-regulation of JNK1/2 and p38 MAPK activation (Jiang et al., 2014). Expression of MAPK/NF-κB signaling proteins, both *in vivo* and *in vitro*, has been evaluated by Hui and Fangyu (2017), who concluded that BB exerts gastroprotective effects *via* the activation of MAPK/NF-κB. In the study of Chen et al. (2017), high glucose-treated human umbilical vein endothelial cells (HUVECs) were subject to various concentrations of GB, and relative p38 MAPK phosphorylation was analyzed by western blot. The results demonstrated that GB can also inhibit p38 MAPK phosphorylation. Furthermore, they found that high glucose-induced expression of TLR4 was inhibited by p38 MAPK inhibitor SB203580. This indicates that p38 MAPK possibly participates in the positive feedback loop with TLR4 signaling and that GB restrains the course (Chen et al., 2017; Figure 3). GB potently inhibited the expression of PF4 and CD40L in thrombin-activated platelets by inhibition of p38 MAPK phosphorylation. So GB might be a promising drug in atherosclerosis through inhibiting platelet function and reducing inflammation (Liu et al., 2014). In addition, GB also exerted anti-inflammatory and chondroprotective effects in LPS-induced chondrocytes by inhibiting LPS-induced MAPK pathway activation, suggesting that GB might be an underlying therapy for osteoarthritis (Hu et al., 2018). Collectively, ginkgolides play a role in the IIR by regulating MAPK signaling pathways, but the detailed mechanisms still need further investigations.

### Toll-Like Receptor/Myeloid Differentiation Primary Response 88/Nuclear Factor-Kappa B Pathway in the Regulation of Inflammatory Immune Response

Myeloid differentiation primary response 88 (MyD88) is an adaptor protein of the toll/IL-1 receptor (TIR) signaling pathway. MyD88 has a TIR domain and can interact with other TIR domains in TIR family cells, mediating downstream signal transduction and playing a key role in TIR signaling pathway (Li and Qin, 2005). NF-κB is a protein that controls transcription of deoxyribonucleic acid, cytokine production, and cell survival and is also a major transcription factor involved with both the innate and adaptive immune response (Smith et al., 2006). Ginkgolides and BB not only inhibited IL-1β, IL-6, IL-8, IL-10, and TNF-α but also attenuated the levels of TLR2, TLR4, MyD88, Bak, and RIP3, which were induced by oxygen-glucose deprivation/reoxygenation (OGD/R) in BV2 microglial cells. Meanwhile, ginkgolides and BB also reduced p-TGF-β-activated kinase 1, p-IkBα, and p-IKKβ and suppressed the OGD/R-induced transfer of NF-κB p65 from the cytoplasm to the nucleus in BV2 microglial cells (Zhou Y. et al., 2016). These results showed that ginkgolides and BB protect BV2 microglial cells against OGD/R injury by inhibiting TLR2/4 signaling pathways (Zhou Y. et al., 2016). The therapeutic effects of GB on ischemic and hemorrhagic stroke are widely recognized. The modulatory effects on inflammatory-related gene expression, suppression of NF-κB and PI3K/Akt pathways, and TLR4/NF-κB are the main protective mechanisms of GB against stroke (Nabavi et al., 2015). Accumulating evidence demonstrated that GB can suppress gene expression of TLR4 and NF-κB; decrease concentrations of inflammatory cytokines such as TNF-α, IL-1β, and IL-6; and reduce the number of apoptotic neuronal cells in both intracerebral hemorrhage rat brain tissue and traumatic brain injury. These all suggested that GB may ameliorate inflammation by suppressing the expression of TLR4-NF-κB signaling pathway (Hu et al., 2011; Yu et al., 2012; Wan et al., 2017). GB also significantly attenuated activation of NF-κB and expression of TNF-α mRNA induced by LPS (Wu et al., 2016). In HUVECs, the expressions of inflammatory protein-intercellular adhesion molecule-1, the activation of IκB phosphorylation, and NF-κB induced by oxidized low-density lipoprotein are all inhibited by GB. The pharmacological effects of GB on the inflammatory response induced by ox-LDL in HUVECs may be associated with its inhibition of NF-κB activation and reduction of reactive oxygen species production (Li et al., 2009). Both GA and GB have ability to inhibit ischemia-induced NF-κB activation by IκBα degradation *via* suppression of the NF-κB-inducing kinase-IκB kinase pathway (Wang et al., 2008). GC also shows a beneficial effect against myocardial I/R injury *via* inhibition of inflammation, possibly *via* suppression of the CD-40-NF-κB signaling pathway and downstream inflammatory cytokine expression. These may offer an alternative treatment for myocardial I/R diseases (Zhang et al., 2018; Figure 3).

The neuroprotective effects of BB may be related to inhibiting the expression of NF-κB p65 protein and decreasing its nuclear translocation in the substantia nigra pars compacta of rats to prohibit the apoptosis of dopaminergic neurons (Li et al., 2008). GB can also protect cultured neurons from hypoxia- and glutamate-induced damage and inhibit neuronal apoptosis by down-regulating pro-apoptotic protein expression including Bcl-2-associated X protein and up-regulating anti-apoptotic protein expression (Ahlemeyer et al., 1999; Gu et al., 2012). The anti-apoptotic property of GB may also contribute to the suppression of p-SAPK/JNK activation and reactive oxygen species, inhibiting mitochondrial pro-apoptotic factors such as caspase-3, caspase-9, poly ADP-ribose polymerase, and cytochrome *c* (Gu et al., 2012). GB is also believed to interfere with the production of free radicals and protect against a decrease in hippocampal Ca^2+^/calmodulin-dependent PKCII activity after cerebral ischemia (Zalewska et al., 1996; Pietri et al., 1997). Moreover, GA and GB decrease glutamate-induced damage of neuronal and hippocampal cells (Prehn and Krieglstein, 1993). Brain-derived neurotrophic factor (BDNF), a member of the neurotrophin family, is present in the mature brain and is implicated to decrease infarct volume and to improve neurological outcomes (Schäbitz et al., 2000, 2007). BDNF activates intracellular tyrosine receptor kinase B, MAPK, and the extracellular signal-regulated kinases to protect against ischemic stroke (Reichardt, 2006). Wei and colleagues found that GB can up-regulate the expression of BDNF in ischemic stroke by evaluating the therapeutic effects of GB in transient middle cerebral artery occlusion mice and OGD/R-treated N2a cells (Wei et al., 2017). Collectively, ginkgolides have anti-PAF, anti-apoptotic, anti-oxidative, neurotrophic, and neuroimmunomodulatory effects.

## Roles of Ginkgolides in The Inflammatory Immune Response of Neurological Diseases

Inflammation and immune response, as an important mechanism, are directly involved in the occurrence of many diseases of the nervous system, such as Parkinson’s disease (PD), ischemic stroke, MS, and Guillain–Barré syndrome (GBS). Ginkgolides might serve as a potential new treatment of these neurological diseases by regulating IIR (Table 2).

**Table 2:** Roles of ginkgolides in IIR in neurological diseases.

Diseases	Actions	Ingredients	References
Neurodegenerative diseases	Inhibition of the NF-κB.	BB	Li et al. (2008)
Ischemic stroke	Decreases infarct size, serum levels of pro-inflammatory factors, expressions of intercellular adhesion molecule-2 and E-selection. Down-regulates TLR4 and NF-κB. Reduces microglial activation and promotes microglia/macrophage transferring from inflammatory M1 phenotype to M2 phenotype. Suppresses ERK/MAPK pathway and inhibits Akt phosphorylation.	GB	Gu et al. (2012)
	Reduces microglial activation and promotes microglia/macrophage transferring from inflammatory M1 phenotype to M2 phenotype.	GB	Gu et al. (2012)
	Suppresses ERK/MAPK pathway and inhibits Akt phosphorylation.	GB	Nabavi et al. (2015)
Neuroimmune diseases	Attenuates the inflammatory responses.	GB	Zhou J.-M. et al. (2016)
	Inhibits the expressions of TLR4 and MyD88.	GB	Chen et al. (2017)
	Regulates the TLR/MyD88/NF-κB.	Ginkgolides	Tran et al. (2018)

*IIR, inflammatory immune response; NF-κB, nuclear factor-kappa B; BB, bilobalide; GB, ginkgolide B; TLR, toll-like receptor; ERK, extracellular regulated protein kinase; MAPK, mitogen-activated protein kinase; MyD88, myeloid differentiation factor 88*.

### Ginkgolides in Parkinson’s Disease

PD is a common neurodegenerative disorder of the central nervous system (CNS), which is characterized by progressive loss of dopaminergic neurons of the substantia nigra pars compacta with a reduction of dopamine concentration in the striatum. The exact PD etiology remains unknown, but a variety of theories attempted to explain the causes of neuronal death and to identify possible triggers. It has been hypothesized that inflammation may underlay the neurodegenerative process, with the immune system playing a key role (Caggiu et al., 2019). A rat model of PD was produced with a unilateral infusion of 6-OHDA into the substantia nigra pars compacta. Different doses of BB were administered to the rat and locomotor activity and rotational behavior, and the expressions of NF-κB were tested after the 6-OHDA infusion. Finally, the study concluded that NF-κB activation contributes to the 6-OHDA-induced loss of dopaminergic neurons, and the inhibition of the NF-κB pathway is likely to be involved in the neuroprotective effect of BB (Li et al., 2008). The roles of ginkgolides in IIR are also supported by the study of Kim et al., wherein they found that GB can significantly attenuate the increase of the NF-κB DNA-binding activity induced by 1-methyl-4-phenyl-1,2,3,6-tetrahydropyridine (can induce PD rodent model through an NF-κB-dependent mechanism; Kim et al., 2013). Furthermore, PD model treatment with GB-nanocrystals (GB-NCs) can improve behavior, reduce dopamine deficiency, and elevate dopamine metabolite levels (Liu et al., 2020). Thus, BB and GB provide a therapeutic approach to rescue the PD by regulating IIR. Highly stabilized GB-NCs had small sizes, high rates of dissolution, and improved oral bioavailability and brain uptake, which might make them effective drugs for anti-PD therapies in the future. But this field is nascent, and further explorations are needed.

### Ginkgolides in Ischemic Stroke

Numerous studies have proven that neuroinflammation plays an important pathological role in ischemic stroke (Chen et al., 2020). Extracellular glutamate increased significantly after ischemia (Hsieh et al., 2017). And this extracellular glutamate can result in microglial activation and production of inflammatory mediators such as pro-inflammatory cytokines, adhesion molecules, and chemokines (Goldshmit et al., 2018). These inflammatory mediators can increase the severity of primary brain damages (Huang et al., 2018). TLR, NF-κB, and nitric oxide also play a crucial role in mediating signaling pathways in microglial activation and ischemic stroke-induced damage (Zheng et al., 2017; Zhao et al., 2019). A growing number of evidence has shown that administration with GB decreased infarct size, serum levels of pro-inflammatory factors (such as TNF-α, IL-6, and IL-1β), and expressions of intercellular adhesion molecule-2 and E-selectin; down-regulated TLR4 and NF-κB; and reduced microglial activation in transient middle cerebral artery occlusion-induced cerebral I/R injury in mice (Gu et al., 2012; Fan et al., 2020). Previous studies also proved that GB promoted microglia/macrophage transferring from inflammatory M1 phenotype to a protective, anti-inflammatory M2 phenotype *in vivo* or *in vitro* (Shu et al., 2016). Other mechanisms have been revealed that the anti-inflammatory activity of GB included suppression of ERK/MAPK pathway, inhibition of Akt phosphorylation, and down-regulation of p-TAK1, p-IkBα, and p-IKKβ (Nabavi et al., 2015; Fan et al., 2020). The effects of ginkgolides and BB in the cellular and signaling events of ischemic stroke, including inflammatory pathways and neuroprotection, have been validated in multiple preclinical studies. In the future, we might focus on the design and synthesis of ginkgolides and BB analogs with brain-targeting ability, which would cause effective and continuous therapy for CNS diseases.

### Ginkgolides in Multiple Sclerosis

MS is a classic neuroinflammatory and immunological disease of the CNS, which is the second major neurological disease leading to the disability of young adults (Hassan-Smith and Douglas, 2011). In the pathogenesis of MS, antigens are ingested and recognized by antigen presenting cells (APCs) such as DCs, which leads to the activation of autoreactive T cells, leading to a series of pathological changes such as CNS inflammation, demyelination of myelin sheath, and destruction of axon (Mundt et al., 2019). DCs can initiate the autoreactive immune response in the pathogenesis of MS and promote and maintain immune tolerance on the other hand (Zhou Y. et al., 2016). PAF is a lipid mediator produced by cell activation, which participates in inflammatory reaction. In the process of inflammation and immune response, immunogen activates a series of signal transduction pathways in cells, which then triggers the expressions of cytokines and participates in inflammations and immune responses. NF-κB and MyD88 are critical intracellular signaling molecules. Recent studies suggested that NF-κB participates in the inflammatory immune responses induced by MΦ. Enhanced NF-κB activity can inhibit the transformation of M1 into M2, so as to strengthen and enlarge inflammation responses and aggravate tissue damages (Vogel et al., 2013). On the contrary, inhibiting the activity of NF-κB can increase the number and function of M2 cells and reduce inflammations and promote the recovery of diseases. MyD88 has a clear relationship with infectious diseases, tumors, and autoimmune diseases; and an MyD88-dependent pathway is considered as a vital target for intervention treatment of these diseases (Feng et al., 2016). TLR/MyD88 signaling pathway is closely related to the maturation of DCs and the secretion of inflammatory cytokines. TLR/MyD88 signaling pathway plays a key role in the pathogenesis of experimental allergic encephalomyelitis (EAE), a classical animal model of MS in human. The onset time of TLR9 knockout mice (TLR9−/−) is delayed compared with that of normal mice, and the clinical symptoms are mild (Prinz et al., 2006).

Ginkgolides regulate the TLR/MyD88/NF-κB signaling pathway and attenuate the inflammatory responses to inhibit the productions of inflammatory factors mediated by OGD/R in microglial cells (Zhou Y. et al., 2016). GB plays a protective role in inhibiting the expressions of TLR4 and MyD88 induced by high glucose and then in alleviating the TLR4-mediated inflammatory responses in endothelial cells (Chen et al., 2017). As shown previously, ginkgolides have been proved to be a PAFR antagonist, significantly reducing the increase of nuclear translocation of NF-κB p65 induced by PCP (Tran et al., 2018). It has been reported that GB plays a role in PAFR antagonist and can effectively prevent synaptic damage in hippocampus of EAE mice without affecting microglial activation (Bellizzi et al., 2016). Recently, Yu et al. (2019) have observed the therapeutic potential of GK in experimental autoimmune neuritis (EAN) through possible cellular and molecular mechanisms, especially as a peripheral immunomodulatory, and provided that GK may be a promising naturally small molecule compound for treatment of MS in the future. Despite that ginkgolide treatment may represent a novel strategy for attenuating the inflammatory responses, the precise mechanism of ginkgolides in mediating IIR remains to be further explored.

### Ginkgolides in Guillain–Barré Syndrome

GBS is an immune-mediated peripheral neuropathy, characterized by demyelination of peripheral nerve and nerve roots and infiltration of small vascular inflammatory cells. EAN is a useful animal model for conducting research on the pathogenesis and treatment of GBS (Liu et al., 2018). A variety of immune cell subsets and a complex network of cytokines are involved in the pathogenesis and progression of GBS/EAN, such as Th1, Th2, Th17, and regulatory T cells (Treg) cells (Zhang et al., 2013). The Th1 response is related to the acute phase response to the pathogen in GBS, whereas the Th2 response is associated with the recovery phase (Zhang et al., 2014). The IFN-γ, IL-6, and TNF-α levels in Th1 are increased in the acute phase of GBS, whereas those of TGF-β and IL-4 are increased during the recovery phase of GBS (Li et al., 2020). Moreover, the proportion of Th17 cells and the levels of IL-17A in the peripheral blood of GBS patients are increased in the acute phase of the disease, and those of IL-17A are related to the disability scale score of GBS (Kharwar et al., 2017). Tregs can abolish antigen-specific T cell proliferation and suppress the secretion of Th1 and Th2 cytokines (Zhang et al., 2013). Previous studies have suggested that Tregs play a critical role in immune responses in autoimmune diseases and that these cell numbers are reduced in patients with GBS and EAN animals, suggesting their crucial role in damage and repair in GBS (Harness and McCombe, 2008). In summary, CD4^+^ T cells exert their effect by releasing effector cytokines, and the net effects of these Th cytokines determine the direction of immune responses and the consequence of GBS/EAN (Harness and McCombe, 2008; Nyati et al., 2011).

MΦ differentiate into two phenotypes after activation: classical activated M1, also known as pro-inflammatory type MΦ, and activated type M2, also known as anti-inflammatory type MΦ (Shapouri-Moghaddam et al., 2018). M1 are involved in the inflammatory damage of myelin sheath through the release of pro-inflammatory factors, such as IL-12, during the early course of GBS (Labonte et al., 2014). M2 are related to disease recovery by secreting anti-inflammatory cytokines in the later stage of GBS (Shen et al., 2018). CD4^+^ T cells and MΦ could interact and promote with each other as the cytokines secreted by them are interconnected, intricate, and pleiotropic. These cytokines constitute a complex immune network in the pathogenesis of GBS/EAN. Previous studies have shown that TLRs play a pivotal role in the occurrence and development of GBS (Nyati and Prasad, 2014). Compared with healthy controls, mRNA levels of TLR2, TLR4, MyD88, and NF-κB were significantly increased in patients with GBS (Du et al., 2015). It was also found that significant up-regulation of TLR2 in sciatic nerves of EAN is correlated with disease severity (Zhang et al., 2009). Moreover, TLR signaling activates antigen presenting cells through MyD88-dependent or MyD88-independent pathways to initiate adaptive immunity (Nyati and Prasad, 2014). Thus, TLR, MyD88, and NF-κB are involved in the pathogenesis of GBS/EAN. Studies have also shown that expression of MyD88 in patients with GBS is increased (Du et al., 2015). We speculated that the roles of ginkgolides in GBS/EAN may be mediated by the regulation of MyD88/NF-κB, based on the fact that ginkgolides attenuate inflammatory responses by regulating the TLR/MyD88/NF-κB signaling pathway.

## Conclusion

Ginkgolides are clinically used for neuroprotective treatment on reconvalescents of cerebral infarction. However, the cognition about its therapeutic mechanism is still lacking. Ginkgolides have several different biological effects including inhibiting platelet aggregation, preventing apoptosis and oxidation, providing nutrition to nerves, and regulating neuroimmunity. Nowadays, accumulating studies have reported that ginkgolides play an important role in regulating IIR *via* inhibiting the PAF-mediated signal transduction, MAPK, and NF-κB signaling pathways, which provide an insight into the novel clinical application of ginkgolides in some neurological disease therapy in the future.

## Author Contributions

CL carried out the literature review and drafted the manuscript. KL, SL, and QA helped to draft the manuscript. XW conceived, designed, and coordinated the study. All authors read and approved the final manuscript.

## Conflict of Interest

The authors declare that the research was conducted in the absence of any commercial or financial relationships that could be construed as a potential conflict of interest.
